# Spatial insurance in multi‐trophic metacommunities

**DOI:** 10.1111/ele.13365

**Published:** 2019-08-08

**Authors:** Romana Limberger, Alexandra Pitt, Martin W. Hahn, Stephen A. Wickham

**Affiliations:** ^1^ Research Department for Limnology University of Innsbruck Mondsee Austria; ^2^ Department of Biosciences University of Salzburg Salzburg Austria

**Keywords:** Bacteria, dispersal, environmental change, experiment, food web, mesocosms, metacommunity, phytoplankton, trophic interactions, zooplankton

## Abstract

Metacommunity theory suggests that dispersal is a key driver of diversity and ecosystem functioning in changing environments. The capacity of dispersal to mitigate effects of environmental change might vary among trophic groups, potentially resulting in changes in trophic interactions and food web structure. In a mesocosm experiment, we compared the compositional response of bacteria, phyto‐ and zooplankton to a factorial manipulation of acidification and dispersal. We found that the buffering capacity of dispersal varied among trophic groups: dispersal alleviated the negative effect of acidification on phytoplankton diversity mid‐experiment, but had no effect on the diversity of zooplankton and bacteria. Likewise, trophic groups differed in whether dispersal facilitated compositional change. Dispersal accelerated changes in phytoplankton composition under acidification, possibly mediated by changes in trophic interactions, but had no effect on the composition of zooplankton and bacteria. Overall, our results suggest that the potential for spatial insurance can vary among trophic groups.

## Introduction

Environments are changing at unprecedented rates, with detrimental effects on biodiversity and ecosystem functioning (Chapin *et al.* 2000). The spatial insurance hypothesis predicts that dispersal can mitigate the negative effect of environmental change on diversity by facilitating species sorting, that is allowing the immigration of species that are adapted to the new environmental conditions (Loreau *et al.* 2003). If the immigrating species are functionally redundant with the species they replace, dispersal may also maintain ecosystem functioning (Loreau *et al.* 2003). Empirical studies have found that the buffering capacity of dispersal depends on the stressor and the ecosystem metric (Thompson & Shurin 2012; Symons & Arnott 2013) and varies widely among studies (Eggers *et al.* 2012; Lindo *et al.* 2012; Thompson & Shurin 2012; Symons & Arnott 2013; de Boer *et al.* 2014). Part of this complexity likely stems from environmental change and dispersal having differential effects on different groups of organisms (Voigt *et al.* 2003; De Bie *et al.* 2012; Guzman *et al.* 2019). Consequently, the capacity of dispersal to mitigate effects of environmental change could vary among functional and trophic groups. Yet, maintenance of diversity at multiple trophic levels is key for maintaining ecosystem multifunctionality (Soliveres *et al.* 2016; Schuldt *et al.* 2018), because different trophic groups can support different ecosystem functions (Soliveres *et al.* 2016).

The importance of spatial insurance could vary among trophic groups because of variation in colonisation ability and in vulnerability to environmental change. Metacommunity theory suggests that the buffering capacity of dispersal increases with dispersal rate, size of the colonising population, availability of suitable species and invasibility of the resident community (Thompson & Gonzalez 2017; Supplementary Material Table S1). These traits and population characteristics could vary systematically among trophic groups. For example, functional groups vary widely in dispersal ability, due to differences in body size, dispersal mode and life history (De Bie *et al.* 2012; Stevens *et al.* 2014). How dispersal ability changes with trophic position is often system‐specific, hampering general predictions on the relationship between trophic level and ability to track environmental change. For example, dispersal ability increases with trophic position in marine pelagic systems (McCann *et al.* 2005), but declines with trophic position in lake communities (Beisner *et al.* 2006). Colonisation success also depends on the population size of the immigrating species (Thompson & Gonzalez 2017), and may thus decline with trophic level because of lower population densities at upper trophic levels (Cohen *et al.* 2003). Moreover, colonisation success depends on the invasibility of the resident community (Thompson & Gonzalez 2017), and thus often increases when resident biomass is reduced (Shurin 2000; Myers & Harms 2009). Consequently, dispersal could have a stronger effect on functional groups that are more vulnerable to environmental change and thus experience a larger loss of resident biomass and diversity. Vulnerability to environmental change tends to increase with trophic position (Petchey *et al.* 1999; Voigt *et al.* 2003), presumably because of smaller population densities, slower growth rates and stronger dependency on other organisms (Purvis *et al.* 2000). Taken together, spatial insurance could be more important at upper trophic levels because of their higher vulnerability to environmental change, but on the other hand, lower colonisation ability might limit the buffering capacity of dispersal at higher trophic levels.

Environmental change and dispersal can both have indirect effects on communities by altering trophic interactions (Gilman *et al.* 2010; Verreydt *et al.* 2012), but this is an underappreciated complexity of the spatial insurance hypothesis. In both aquatic and terrestrial ecosystems, the effect of environmental change on primary producers can indirectly impact organisms at higher trophic levels (Wade *et al.* 2017; Ullah *et al.* 2018), and effects of environmental change on herbivores or predators can cascade down to lower trophic levels (Martin & Maron 2012; Amundrud & Srivastava 2016; Bell *et al.* 2019). The interaction of environmental change and dispersal might entail additional indirect effects. For example, predators may be precluded from tracking environmental change if their prey is unavailable in the novel habitat, which could further reduce the potential for spatial insurance at upper trophic levels (Thompson & Gonzalez 2017).

Variation among trophic levels in vulnerability to environmental change can result in changes in food web and biomass structure. For example, environmental change can alter the strength of top‐down vs. bottom‐up effects (Kratina *et al.* 2012), the flux of biomass through food webs (Ledger *et al.* 2013), and the shape of biomass pyramids (O'Connor *et al.* 2009; de Sassi & Tylianakis 2012). Metacommunity theory predicts that dispersal, by maintaining species diversity, can maintain food web properties such as number of interactions per species and number of trophic levels (Thompson & Gonzalez 2017). However, maintenance of food web structure may be contingent on whether immigrating species perform the same functions as the species they replace. For example, dispersal‐mediated maintenance of biomass structure may require immigration of species that are functionally redundant for prey edibility and grazing efficiency, because these traits influence transfer efficiency among trophic levels and thus the shape of biomass pyramids (McCauley *et al.* 2018). Currently, however, we are lacking empirical studies testing how environmental change and dispersal interactively influence multiple trophic levels and food web properties.

In a mesocosm experiment, we investigated the interactive effects of environmental change and dispersal on the diversity, composition and biomass structure of multi‐trophic communities. Experimental aquatic ecosystems were either unstressed or exposed to acidification, and were either isolated from or connected to the regional species pool. We expected that dispersal would alleviate the negative effect of environmental change on diversity and facilitate species sorting, as predicted by the spatial insurance hypothesis (Loreau *et al.* 2003) and by multitrophic metacommunity theory (Thompson & Gonzalez 2017). We tested these predictions with three groups of organisms (bacteria, phytoplankton and macrozooplankton), and hypothesised that trophic groups would differ in whether dispersal mitigates the negative effect of environmental change on diversity (Hypothesis 1) and facilitates compositional change (Hypothesis 2). We expected that acidification would have a more negative effect on the diversity of the top trophic level (i.e. macrozooplankton), resulting in higher importance of spatial insurance for zooplankton than for bacteria and phytoplankton. Alternatively, smaller population sizes and lower growth rates of immigrating zooplankton could result in weaker effects of dispersal compared with bacteria and phytoplankton. We also tested the hypothesis that dispersal maintains the biomass and size structure of food webs exposed to environmental change (Hypothesis 3). We predicted that negative effects of acidification on zooplankton biomass would translate in indirect positive effects on phytoplankton biomass, resulting in reduced consumer:resource ratios (i.e. reduced top‐heaviness of the biomass pyramid). Building on the spatial insurance hypothesis (Loreau *et al.* 2003), we expected that dispersal would restore the biomass structure of the food web if it resulted in the immigration of functionally redundant species. To evaluate if dispersal maintained functions that potentially influence biomass structure, we quantified treatment effects on phytoplankton edibility and mean individual zooplankton biomass as proxy for grazing efficiency (Peters & Downing 1984).

We found that the buffering capacity of dispersal varied among trophic groups, with strongest evidence of spatial insurance observed for phytoplankton. Relaxed top‐down control under environmental change likely contributed to the pronounced effect of dispersal on the phytoplankton community. Collectively, our results highlight the importance of integrating multi‐trophic interactions into metacommunity ecology in general, and into the spatial insurance hypothesis in particular.

## Materials and Methods

### Experiment

Sixteen mesocosms were set up in a randomised block design in the Botanical Garden of the University of Salzburg. At the start of the experiment (May 13, 2014), we filled the mesocosms (300 L rain barrels) with unchlorinated tap water and inoculated each barrel with 50 L pond water. The inoculum was a mixture of water from five different ponds and lakes (Table S2). Large predators such as *Chaoborus* larvae were removed from the inoculum, and the mesocosms were covered with a nylon mesh to prevent colonisation by macroinvertebrates. Before we started the experimental treatments, we left the mesocosms for five weeks to establish plankton communities. During the establishment phase, mesocosms received nutrients twice a week (10 µg L^−1^ P, 160 µg L^−1^ N, 160 µg L^−1^ Si).

In a 2 × 2 factorial design, we manipulated environmental change (ambient or low pH) and connectivity (with or without dispersal) to the regional species pool (i.e. 35 aquatic ecosystems). The four treatment combinations were replicated in four blocks. The goal of the environmental change treatment was to expose communities to increasingly stressful conditions. To this end, we reduced the pH value to 4.5 in half of the mesocosms by daily additions of hydrochloric acid. From day 6 to day 37 we gradually reduced the pH value in 0.1–0.2 intervals and then maintained the mesocosms at pH 4.5 until the end of the experiment (day 140) (Fig. S1a). A pH of 4.5 is far outside the pH of the five ponds used for initial inoculation of the mesocosms (Table S2), and thus presumably stressful for the experimental communities.

To simulate connectivity to the regional species pool, we regularly inoculated half of the mesocosms with water from ~ 35 ponds and lakes (Table S3). Immigration events were applied six times over the 20‐week course of the experiment (on days 8, 30, 51, 62, 94 and 120; Fig. S1a). At each immigration event, we collected 4 L of water each from 34–35 water bodies around the city of Salzburg. The pH value in the immigration ponds and lakes ranged from 4.2 to 8.8, with the majority of water bodies having pH values around 8 (median: 7.98; Fig. S2). Since we did not want to add an additional trophic level in mesocosms with dispersal, we filtered the immigration water through a mesh of 100 µm mesh size and manually removed predatory macroinvertebrates from the fraction of > 100 µm before mixing all collected water in a rain barrel. We then added 6 L of immigration water to half of the mesocosms, that is 2% of mesocosm water was exchanged with immigration water. To account for possible effects on water chemistry, we also added 6 L of immigration water to unconnected mesocosms, but after removal of organisms by filtration through 0.2 µm. We cannot exclude that unconnected mesocosms received airborne immigration, but we are confident that our dispersal manipulation resulted in strong differences in dispersal rate among mesocosms with and without dispersal respectively.

The immigration treatment resulted in an addition of on average 1,253 individuals of Crustacea (including Nauplia and Copepodits) per immigration event, representing an average addition of 3% of total macrozooplankton present in the mesocosms (Fig. S3). Over the course of the six immigration events, 23 Crustacean taxa were added that were never observed in unconnected tanks. Of these 23 taxa, six established successfully in at least one of the connected mesocosms. In phytoplankton, the dispersal treatment added on average 617 × 10^6^ cells per immigration event and included 108 new taxa that were never observed in unconnected tanks, nine of which established in connected tanks. Many phytoplankton taxa were not identified to species level, resulting in an underestimation of regional richness. Although we exchanged the same amount of water volume (2%) for phyto‐ and zooplankton, immigration rate of phytoplankton was usually higher (Fig. S3) because of the often low phytoplankton abundance in the mesocosms. However, the phytoplankton taxa that numerically dominated the dispersal pool did either not establish in the mesocosms or were part of the resident community (Table S4 and Fig. S4).

### Sampling

The experiment lasted for 20 weeks (June 17 to November 5, 2014), until temperatures started to decline (Fig. S1b). Mesocosms were sampled at seven occasions (on days 0, 27, 43, 56, 84, 113 and 140). We measured abiotic parameters at all sampling dates, composition and abundance of phyto‐ and macrozooplankton and abundance of ciliates at four sampling dates (days 0, 56, 84 and 140), and composition of bacteria at three sampling dates (days 56, 84 and 140).

At sampling events, we mixed the water in each mesocosm with a paddle and removed 15 L of water from each mesocosm by taking eleven integrated samples at randomly selected positions using plexiglass tubes. We fixed 150 mL water with Lugol’s solution for quantification of phytoplankton composition and ciliate abundance. For quantification of Crustacean zooplankton, we filtered 7 L water through a 30 µm mesh and fixed the sample 1 : 1 with a Formol‐Sucrose solution. For analysis of bacterial community composition, we filtered 150‐300 mL of water onto 0.2 µm filters (Whatman Nuclepore Track‐Etch filters) and stored the filters at −70 °C until further processing. We measured pH and temperature with a hand‐held probe.

### Sample processing

To determine phytoplankton composition, we counted samples under an inverted microscope. Taxa were identified to species level when possible, but usually to genus or morpho‐taxon level. To quantify phytoplankton biovolume, a proxy of biomass, we measured up to 10 individuals per taxon, and estimated cellular biovolume following Hillebrand *et al. *(1999). For less abundant taxa we used published biovolume data (Kremer *et al.* 2014) or estimated biovolume based on taxa with similar size in our samples. To determine the composition of macrozooplankton (i.e. Crustacea), we counted zooplankton samples under a stereomicroscope. Cladocera were identified to species level, whereas Copepoda were differentiated into Cyclopoida and Calanoida. To estimate zooplankton biomass, we measured the lengths of ~ 30 individuals per taxon and calculated biomass based on published length‐dry weight relationships (Dumont *et al.* 1975; Bottrell *et al.* 1976; Culver *et al.* 1985; Baumgärtner & Rothhaupt 2003).

Community composition of bacteria was quantified using amplified ribosomal intergenic spacer analysis (ARISA), a molecular fingerprinting technique based on size differences of the intergenic transcribed spacer (ITS) region. We extracted DNA from filters with enzymatic digestion and phenol‐chloroform‐isoamylalcohol (Gich *et al.* 2005). The ITS region was amplified with 6‐FAM‐labelled universal forward primer 1406f and bacteria‐specific reverse primer 23Sr (modified from Yannarell *et al.* 2003). After purification of PCR products (GeneJET PCR purification kit, Thermo Scientific), samples were analysed with denaturing gel electrophoresis on a MegaBACE 1000 (GE Healthcare Biosciences, Pittsburgh, PA, USA), with ROX‐labelled MapMarker 1500 (BioVentures) used as internal size standard. All samples were measured in the same run. Fragment sizes and relative peak areas were determined with GeneMarker V2.6.4 (SoftGenetics) using the local southern method for size calling. The peaks were binned with interactive binner v1.4 (Ramette 2009), using a window size of 2 bp. We included peaks in the size range of 100 to 1000 bp with a relative fluorescence intensity over 0.2%.

### Data analysis

We analysed treatment effects on the diversity and composition of bacteria, phyto‐ and zooplankton (Hypotheses 1 and 2) and on the biomass and size structure of communities (Hypothesis 3). To calculate the ratio of zooplankton to phytoplankton biomass, we converted phytoplankton biovolume to biomass using a conversion factor of 1 (Yuan & Pollard 2018), that is 10^6^ µm^3^ are 1 µg. To investigate treatment effects on zoo‐ and phytoplankton size structure, we computed the proportion of edible phytoplankton (i.e. phytoplankton with cell or colony size < 30 µm; Bell 2002) and mean individual macrozooplankton biomass.

All analyses were computed with R version 3.4.1. We used the package *lme4* to calculate linear mixed models with pH and dispersal as fixed factors and block as random factor to test for treatment effects on diversity, biomass and size structure. We computed *P*‐values with the *car* package using type II Wald F tests with Kenward–Roger approximation for the degrees of freedom. The level of significance was adjusted by Bonferroni‐correction (four time points; α = 0.0125). Time series data were analysed with linear mixed models with day, dispersal and pH as fixed factors, and block and mesocosm as random factors to account for repeated sampling of mesocosms. However, since treatment effects often interacted with time (Table S5), we here focus on the results for individual sampling dates. To test for treatment effects on the composition of bacteria, phyto‐ and zooplankton, we used the package *vegan* to compute partial distance‐based redundancy analysis (db‐RDA) of Hellinger‐transformed biomass data, using Euclidean distance as distance measure, pH and dispersal as constraining variables and block as conditional variable. We ran 10 000 random permutations to calculate *P*‐values using the function anova.cca in *vegan*. We also computed unconstrained multivariate analyses using principal coordinate analysis (PCoA), and used those to visualise treatment effects on community composition.

## Results

### Diversity and composition

**Figure 1:**
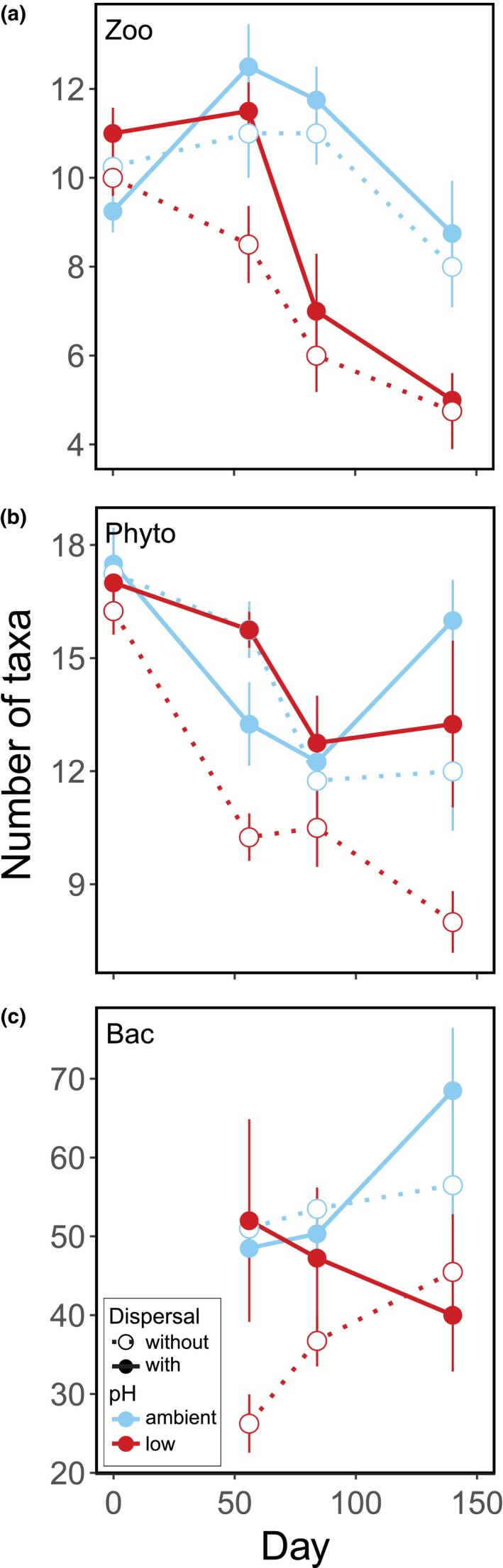
Effects of pH and dispersal on the diversity of (a) macrozooplankton (Crustacea), (b) phytoplankton and (c) bacteria. Dotted line and open symbols denote treatments without dispersal, solid line and filled symbols denote treatments with dispersal. Ambient pH treatments are blue, low pH treatments are red. Values are means ± SE, *n* = 4.

The capacity of dispersal to alleviate negative effects of acidification on diversity varied among trophic groups and time points (Table 1 and Fig. 1). Dispersal had no effect on zooplankton diversity at the Bonferroni‐adjusted level of significance (Table 1 and Fig. 1a). Dispersal mitigated the negative effect of acidification on phytoplankton diversity on day 56 (Table 1 and Fig. 1b), increasing diversity by 55% (± 8; SE) at low pH. At the end of the experiment, dispersal increased phytoplankton diversity irrespective of pH (Table 1 and Fig. 1b), by 39 % (± 14) at ambient pH and by 65 % (± 19) at low pH. Bacterial diversity was unaffected by dispersal (Table 1 and Fig. 1c). Similarly, when comparing effect sizes of dispersal at low pH among groups, we found significant effects of dispersal only for phytoplankton diversity (days 56 and 140; Fig. S5).

**Table 1:** Results (i.e. parameter estimates and *P*‐values) of linear mixed models testing for effects of pH and dispersal on diversity and biomass structure. Transformations are listed below the response variables. Bold font denotes *P*‐values < 0.0125 (accounting for four comparisons), italic font denotes *P*‐values < 0.05

Response	Day	Intercept	pH		Dispersal		pH × Dispersal	
		Estimate	Estimate	*P*	Estimate	*P*	Estimate	*P*
Zoo diversity	0	10.25	−0.25	0.117	−1.00	1.000	2.00	*0.046*
	56	11.00	−2.50	0.077	1.50	*0.031*	1.50	0.415
	84	11.00	−5.00	**<0.001**	0.75	0.367	0.25	0.895
	140	8.00	−3.25	**0.003**	0.75	0.585	−0.50	0.784
Phyto diversity	0	17.25	−1.00	0.386	0.25	0.558	0.50	0.768
	56	15.75	−5.50	0.086	−2.50	0.086	8.00	**0.001**
	84	11.75	−1.25	0.777	0.50	0.312	1.75	0.513
	140	12.00	−4.00	*0.019*	4.00	**0.004**	1.25	0.611
Bac diversity	56	51.00	−24.75	0.149	−2.50	0.119	28.25	0.066
	84	53.50	−16.75	0.083	−3.17	0.440	13.67	0.242
	140	56.50	−11.00	*0.019*	12.00	0.652	−17.50	0.240
Zoo biomass	0	6.49	0.21	0.385	0.01	0.896	−0.07	0.848
ln(y)	56	6.10	0.02	*0.047*	−0.22	0.510	0.66	0.059
	84	6.06	−0.38	0.841	−0.18	0.665	0.63	0.341
	140	5.75	0.05	0.861	0.14	0.659	0.02	0.972
Ciliate abundance	0	1.17	0.38	0.372	0.28	0.568	−0.25	0.660
ln(y)	56	2.16	−1.32	0.559	0.36	**0.003**	3.23	**0.009**
	84	3.14	−0.87	0.473	−0.38	0.194	2.87	0.088
	140	3.62	−0.87	0.825	−0.32	0.605	1.41	0.350
Phyto biovolume	0	0.23	−0.03	0.977	−0.08	0.370	0.06	0.497
ln(y)	56	0.44	0.58	**<0.001**	−0.07	*0.022*	−0.28	0.094
	84	0.28	0.33	**<0.001**	−0.06	**0.002**	0.93	**0.001**
	140	0.37	1.12	**0.002**	0.01	0.997	−0.01	0.978
Zoo: Phyto	0	0.43	0.14	0.692	0.18	0.532	−0.14	0.687
log_10_(y)	56	−0.09	−0.49	*0.042*	0.00	*0.039*	0.50	*0.037*
	84	0.20	−0.64	**<0.001**	−0.01	0.164	−0.41	0.185
	140	−0.14	−0.82	**0.011**	0.07	0.740	0.02	0.962
Ind Zoo biom	0	1.03	0.38	*0.030*	0.24	0.272	−0.23	0.281
ln(y)	56	1.07	−0.26	**0.006**	−0.02	0.377	−0.12	0.511
	84	1.07	−0.58	0.143	−0.01	0.118	0.61	0.110
	140	0.98	−0.35	0.320	−0.05	*0.041*	1.18	*0.030*
Edible Phyto	0	1.20	−0.05	0.919	−0.09	0.624	0.11	0.472
arcsin(sqrt(y))	56	1.03	0.47	**<0.001**	−0.01	0.271	−0.10	0.371
	84	0.90	0.53	**<0.001**	0.09	0.382	−0.01	0.942
	140	1.08	0.47	**<0.001**	−0.05	0.428	0.02	0.829

Bac, Bacteria; Edible phyto, proportion of edible phytoplankton; Ind Zoo Biom, mean individual zooplankton biomass; Phyto, Phytoplankton; Zoo, Zooplankton; Zoo:Phyto, ratio of zooplankton to phytoplankton biomass.

**Figure 2:**
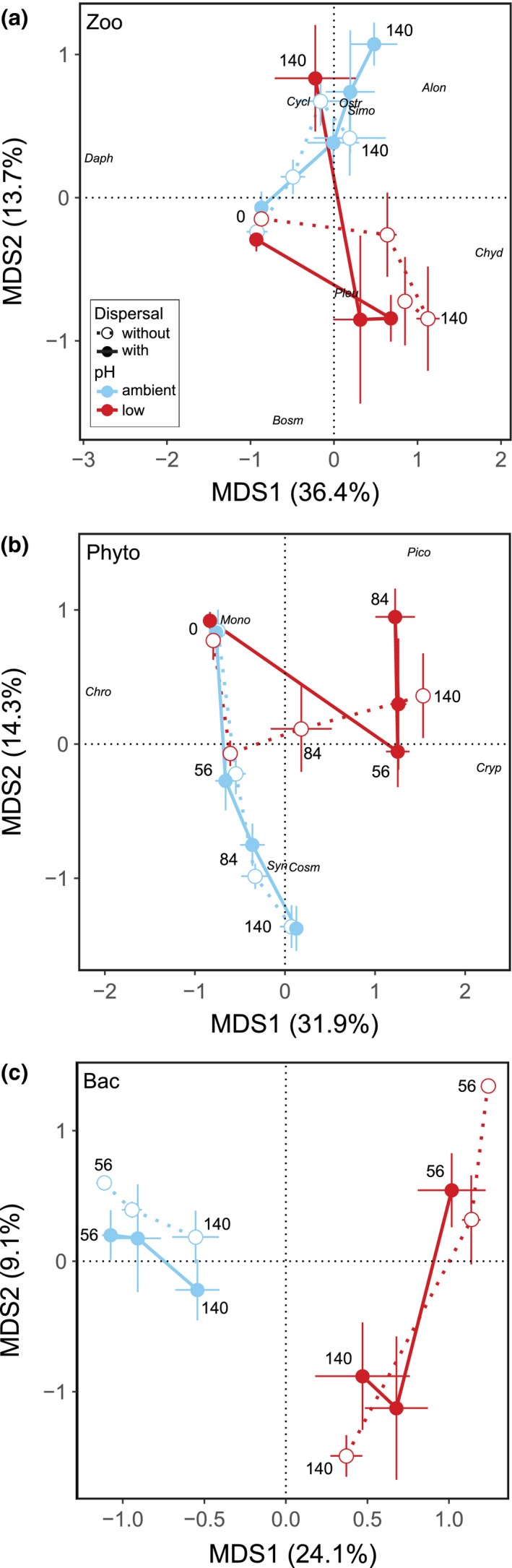
Principal coordinates analyses (PCoA) of community composition. PCoA was computed with (a) macrozooplankton biomass, (b) phytoplankton biovolume and (c) relative peak area of bacteria. Mean sample scores are connected by date, with the first and last sampling day labelled. Taxa with highest loadings are displayed for (a) zooplankton (Alon: *Alona affinis*, Bosm: *Bosmina longispina*, Chyd: *Chydorus sphaericus*, Cycl: *Cyclopoida*, Daph: *Daphnia pulex*, Ostr: *Ostracoda*, Pleu: *Pleuroxus truncatus*, Simo: *Simocephalus vetulus*) and (b) phytoplankton (Chro: *Chromulina*, Cosm: *Cosmarium*, Cryp: *Cryptomonas*, Mono: *Monoraphidium*, Pico: *Picococcales*, Syn: *Synechococcus*). Values are means ± SE, *n* = 4.

Community composition of zoo‐, phytoplankton and bacteria differed between the two pH levels, whereas the importance of dispersal varied among trophic groups and time points (Table 2 and Fig. 2). Zooplankton composition differed between ambient and low pH mid‐experiment (Table 2 and Fig. 2a), with higher relative biomass of the large Cladoceran *Daphnia pulex* at ambient pH and higher relative biomass of the small Cladoceran *Chydorus sphaericus* at low pH (Fig. S6a). Towards the end of the experiment, *C. sphaericus* remained dominant in low‐pH mesocosms without dispersal, but declined in biomass in low‐pH mesocosms with dispersal (Figs 2a and S6a). Instead, larger Crustaceans established on day 140 in the treatment combination with dispersal and low pH, but their identity varied among replicates: two replicates were dominated by *D. pulex*, whereas two replicates were dominated by copepods. However, treatment effects on overall zooplankton composition were not significant at the end (Table 2). Phytoplankton composition was interactively affected by dispersal and pH on day 56 (Table 2 and Fig. 2b), when phytoplankton composition in connected, low‐pH mesocosms diverged from all other treatment combinations, in line with the prediction of dispersal facilitating species sorting. On day 56, ambient and unconnected low‐pH mesocosms were dominated by small chrysoflagellates (*Chromulina*), whereas connected low‐pH mesocosms were dominated by *Cryptomonas* (Figs 2b and Fig. S6b). However, the interactive effect of dispersal and pH was transient, appearing only on day 56 (Table 2); later in the experiment, phytoplankton composition in low‐pH mesocosms converged to similar composition irrespective of dispersal (Fig. 2b). Composition of bacteria differed among low and ambient pH, but was unaffected by dispersal (Table 2 and Fig. 2c).

**Table 2:** Results of db‐RDA testing for effects of pH and dispersal on the composition of zooplankton, phytoplankton and bacteria. Analyses were based on Hellinger‐transformed biomass data of zoo‐ and phytoplankton, and Hellinger‐transformed relative peak area of bacteria. The entries in the table are the *P*‐values and the proportion of variance explained by pH and dispersal respectively. *P*‐values were calculated with 10 000 permutations. Bold font denotes *P* < 0.0125

	Day	pH	Dispersal	pH × Dispersal	Variance explained
Zooplankton	0	0.498	0.828	0.334	14.0
	56	**< 0.001**	0.128	0.065	47.6
	84	**< 0.001**	0.264	0.088	37.8
	140	0.356	0.132	0.090	31.3
Phytoplankton	0	0.706	0.481	0.302	15.2
	56	**< 0.001**	**< 0.001**	**0.001**	62.2
	84	**< 0.001**	0.199	0.083	44.1
	140	**0.002**	0.579	0.564	45.6
Bacteria	56	**< 0.001**	0.189	0.205	55.8
	84	**< 0.001**	0.165	0.254	43.7
	140	**0.002**	0.802	0.519	29.0

### Biomass and size structure

**Figure 3:**
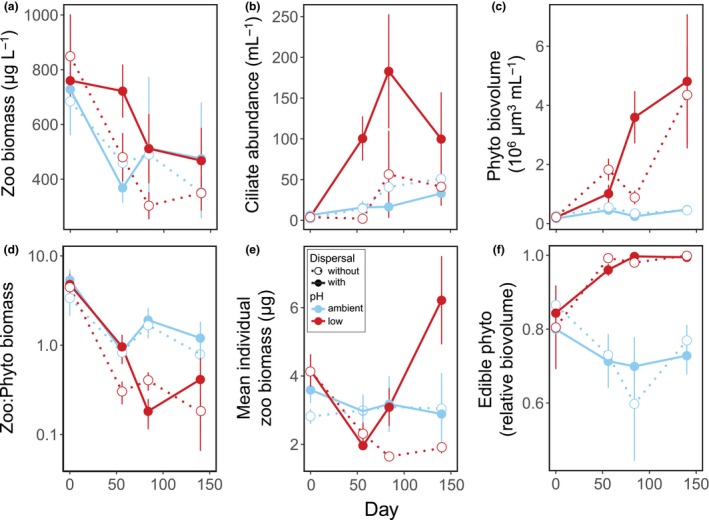
Biomass and size structure of the food web. Effects of pH and dispersal on (a) biomass of macrozooplankton (Crustacea), (b) ciliate abundance, (c) phytoplankton biovolume, (d) the ratio of zooplankton to phytoplankton biomass (note log‐scale of *y*‐axis), (e) mean individual macrozooplankton biomass and (f) the proportion of edible phytoplankton. Values are means ± SE, *n* = 4.

Acidification changed the size structure of phyto‐ and zooplankton and influenced how biomass was distributed among trophic groups; dispersal altered some of the effects on biomass, but did not restore the biomass structure observed at ambient pH (Table 1 and Fig. 3). Neither pH nor dispersal influenced the total biomass of macrozooplankton (i.e. Crustacea) (Table 1 and Fig. 3a). In contrast, dispersal increased the abundance of microzooplankton (i.e. ciliates) at low pH on day 56 (Table 1 and Fig. 3b), with 71 (± 29) times higher ciliate abundance in connected than unconnected low‐pH mesocosms. Acidification had a positive effect on phytoplankton biomass throughout the experiment, which was amplified by dispersal on day 84 (Table 1 and Fig. 3c), with 5 (± 1) times higher phytoplankton biomass in connected than unconnected low‐pH mesocosms. The positive effect of acidification on phytoplankton biomass translated into a reduced ratio of zooplankton:phytoplankton biomass (i.e. reduced top‐heaviness) on days 84 and 140, irrespective of dispersal (Table 1 and Fig. 3d). Acidification changed zooplankton size structure to dominance of smaller taxa on day 56, reflected in lower average individual biomass (Table 1 and Fig. 3e). At the end of the experiment, however, dispersal tended to reverse the negative effect of acidification on average individual zooplankton biomass (Fig. 3e), with 3.4 (± 0.7) times higher individual biomass in connected than unconnected low‐pH mesocosms, but this effect was not significant at the Bonferroni‐adjusted significance level (Table 1). The proportion of edible (i.e. small) phytoplankton increased in response to acidification, irrespective of dispersal (Table 1 and Fig. 3f).

## Discussion

We found that the capacity of dispersal to mitigate negative effects of acidification on diversity differed among trophic groups. Dispersal maintained phytoplankton diversity at undisturbed levels mid‐experiment and generally increased phytoplankton diversity at the end, but had no effect on the diversity of zooplankton and bacteria. Similarly, the capacity of dispersal to facilitate compositional change varied among trophic groups. Dispersal altered community composition of phytoplankton mid‐experiment at low pH, whereas the compositional response of zooplankton was less pronounced, possibly reflecting differences in life history and invasibility of the resident community. The capacity of dispersal to restore biomass and size structure of food webs was limited. Acidification shifted the zooplankton community to dominance of smaller taxa mid‐experiment, and increased phytoplankton biomass and the proportion of edible phytoplankton, suggesting reduced top‐down control at low pH. Dispersal only partly restored food web structure, promoting the re‐establishment of large herbivores, but having no effect on consumer:resource ratios.

The response of the phytoplankton community followed predictions of the spatial insurance hypothesis mid‐experiment (Loreau *et al.* 2003). On day 56, connectivity to the regional species pool maintained diversity of acidified phytoplankton communities at undisturbed levels and accelerated species sorting in response to acidification (Figs 1b and 2b). Later in the experiment, however, the effect of dispersal on phytoplankton composition disappeared, possibly reflecting airborne immigration of phytoplankton in tanks without assisted immigration or seasonal forcing because of low temperatures (Fig. S1b). The more pronounced effect of dispersal on phyto‐ than on zooplankton might be explained by the faster growth rate of phytoplankton, higher numbers of immigrating organisms, higher immigration rates, a more diverse regional species pool, and/or higher invasibility of the resident community. In the early stage of the experiment, the immigration rate was higher in phyto‐ than in zooplankton (25 vs. 2%; Fig. S3), although the exchange rate of mesocosm water was the same for all trophic groups (2%). The high immigration rate in phytoplankton was a consequence of the low phytoplankton abundance in the mesocosms relative to the phytoplankton abundance in the regional species pool. The strong effect of dispersal on phytoplankton diversity and composition on day 56 could be an indication of strong mass effects resulting from high immigration rates. However, at the immigration event prior to day 56, only 3% of the immigrating individuals were of taxa that were responsible for the diversity response on day 56 (Table S4). Similarly, *Cryptomonas*, the major driver of the compositional response on day 56, immigrated with low numbers; its abundance in connected, low‐pH mesocosms on day 56 was on average 79 times higher than its abundance in the immigration pool at the preceding immigration event. We thus conclude that the observed effects of dispersal on phytoplankton diversity and composition did not result from high numbers of immigrants swamping the local community, but from successful establishment of viable populations.

High invasibility of the resident community, mediated by changes in trophic structure, could be an additional reason for the strong effect of dispersal on the phytoplankton community mid‐experiment. As long as top‐down control was maintained (i.e. at ambient pH), dispersal had little effect on phytoplankton despite high immigration rates. Similarly, previous studies found no effect of dispersal when competitors or predators reduced the establishment success of immigrating organisms (Shurin 2000; Howeth & Leibold 2010). However, mid‐experiment, when acidification had reduced large herbivores (Fig. 3e), dispersal resulted in a pronounced restructuring of the phytoplankton community and in a strong increase in phytoplankton biomass (Figs 2b and 3c). Possibly, the combination of strong top‐down control prior to acidification and reduced top‐down control under acidification resulted in high invasibility of the phytoplankton community because of both low resident phytoplankton biomass and low grazing pressure. Collectively, these results suggest that trophic interactions mediated the strong interactive effect of acidification and dispersal on phytoplankton mid‐experiment by influencing the invasibility of the resident community. To test this hypothesis more directly, a factorial manipulation of environmental change, dispersal and food chain length would be an interesting avenue in future experiments.

The macrozooplankton community was primarily structured by environmental change and largely unaffected by dispersal. In unconnected ecosystems, acidification decreased the abundance of large herbivores (e.g. *Daphnia pulex*), and increased the abundance of smaller crustaceans (e.g. *Chydorus sphaericus*), in line with results from whole‐lake acidification studies (Klug *et al.* 2000; Vinebrooke *et al.* 2003). In connected ecosystems, large herbivores re‐established at low pH at the end of the experiment, but their identity varied among replicates: two mesocosms were dominated by copepods, two by *Daphnia pulex*. Low dispersal rates and a large regional species pool possibly contributed to the variation in community composition among replicate ecosystems (Chase 2003). We had hypothesised that spatial insurance could be more important in zoo‐ than in phytoplankton because high vulnerability of zooplankton to environmental change could lead to high invasibility of the resident zooplankton community. In contrast to this prediction, however, dispersal was less important in zooplankton than in phytoplankton. It seems that acidification did not increase invasibility of the zooplankton community, as small resident taxa outweighed the decline in large herbivores. The high resident zooplankton biomass, in combination with slower growth rates and a lower absolute number of immigrants likely resulted in reduced establishment success of immigrating zooplankton relative to phytoplankton. We cannot discern between these mechanisms, but it seems that trophic position along the food chain was the major driver of resident biomass and thus at least partly determined if local or regional processes prevailed once the environment changed.

Environmental change reduced bacterial diversity and altered bacterial community composition, but dispersal had no effect overall. Previous studies found little evidence of dispersal‐limitation in bacteria (Beisner *et al.* 2006; De Bie *et al.* 2012), but also showed that dispersal can facilitate recovery of bacterial communities after perturbations (Baho *et al.* 2012). We found no evidence of dispersal promoting species sorting in bacteria, maybe indicating that the regional species pool did not contain suitable taxa, possibly because of different environmental conditions in natural low‐pH ponds and low‐pH mesocosms. Environmental change often results in novel combinations of environmental conditions (Visser 2008), precluding establishment of immigrating species. Accordingly, differences in chemistry (e.g. in concentrations of ions and humic substances) between low‐pH natural ponds and low‐pH mesocosms might have inhibited successful establishment of bacterial taxa from the regional species pool. Alternatively, the lack of dispersal effect could be the result of a comparatively coarse taxonomic resolution of the molecular fingerprinting approach that we used to determine bacterial community composition. ARISA differentiates among taxa that differ in the length of the ITS region, but bacterial taxa with different ecological requirements can have similar lengths of the ITS region (Hahn *et al.* 2016). Possibly, ARISA detected the strong impact of acidification, but not the potentially finer‐scale effects of dispersal. In future experiments, quantifying bacterial diversity with next generation sequencing could help detect such fine‐scale differences (Limberger *et al.* 2017).

The capacity of dispersal to restore the biomass and size structure of food webs exposed to acidification was limited. Unstressed ecosystems were characterised by dominance of large herbivores, in particular mid‐experiment, and low phytoplankton biomass, indicating strong top‐down control. Acidification shifted the zooplankton size structure to dominance of smaller taxa that were less efficient at reducing phytoplankton, as indicated by increased phytoplankton biomass, a larger proportion of edible phytoplankton, and reduced zooplankton:phytoplankton ratios (Fig. 3). Dispersal amplified the effect of acidification on phytoplankton biomass mid‐experiment, but tended to reverse the effect of acidification on zooplankton size structure at the end, promoting re‐establishment of large herbivores (Fig. 3e). However, the capacity of dispersal to fully restore top‐down control varied among replicates and depended on the identity of the herbivores that established. Phytoplankton biomass only declined again when *Daphnia pulex*, an efficient herbivore, re‐established (Fig. S7d), but not when dispersal resulted in dominance of copepods. We had predicted that dispersal would maintain the biomass structure of food webs exposed to environmental change if dispersal resulted in the immigration of species that perform similar functions as the species they replace. However, our findings highlight that species are not necessarily redundant in how they control ecosystem processes (Cardinale *et al.* 2006), and the capacity of dispersal to maintain food web structure may thus be limited when trophic interactions are strongly determined by single keystone species.

In summary, we found that the buffering capacity of dispersal varied among trophic groups, presumably because (1) groups of organisms varied in life‐history traits and population characteristics and (2) because interactions among trophic groups influenced the establishment success of immigrating species. Our experiment does not entail the conclusion that spatial insurance will generally be more important in primary producers than in higher trophic levels. Rather, our results suggest that the buffering capacity of dispersal will be largest in those trophic levels that have high colonisation ability, and/or are released from top‐down control when the environment changes. Which trophic level that is, however, will depend on ecosystem type and food web structure. For example, aquatic and terrestrial ecosystems vary in how dispersal ability changes with trophic level (De Bie *et al.* 2012; Stevens *et al.* 2014). Similarly, ecosystems vary widely in food web properties, such as food chain length (Ward & McCann 2017) and the strength of bottom‐up versus top‐down forces (Gripenberg & Roslin 2007). Such variation in food web architecture likely influences the importance of trophic interactions in mediating effects of spatial insurance. In general, however, evidence is mounting that indirect effects of environmental change via altered trophic interactions are as important as the direct effects, in both aquatic and terrestrial ecosystems (Tylianakis *et al.* 2008; Ockendon *et al.* 2014). Integrating the complexities that arise from trophic interactions into metacommunity ecology will thus be of fundamental importance for predicting the response of complex, multitrophic communities to environmental change.

## Authorship

RL designed the experiment. RL and AP implemented the experiment. All authors contributed to sample analyses. RL analysed the data and wrote the manuscript. All authors provided comments on the manuscript.

## Data Availability

Data available from Dryad Digital Repository: https://doi.org/10.5061/dryad.360k8v0.

## References

[ele13365-bib-0001] Amundrud, S.L. & Srivastava, D.S. (2016). Trophic interactions determine the effects of drought on an aquatic ecosystem. Ecology, 97, 1475–1483. 27459778 10.1890/15-1638.1

[ele13365-bib-0002] Baho, D.L. , Peter, H. & Tranvik, L.J. (2012). Resistance and resilience of microbial communities ‐ temporal and spatial insurance against perturbations. Environ. Microbiol., 14, 2283–2292. 22513226 10.1111/j.1462-2920.2012.02754.x

[ele13365-bib-0003] Baumgärtner, D. & Rothhaupt, K.O. (2003). Predictive length‐dry mass regressions for freshwater invertebrates in a pre‐alpine lake littoral. Int. Rev. Hydrobiol., 88, 453–463.

[ele13365-bib-0004] Beisner, B.E. , Peres‐Neto, P.R. , Lindström, E.S. , Barnett, A. & Longhi, M.L. (2006). The role of environmental and spatial processes in structuring lake communities from bacteria to fish. Ecology, 87, 2985–2991. 17249222 10.1890/0012-9658(2006)87[2985:troeas]2.0.co;2

[ele13365-bib-0005] Bell, T. (2002). The ecological consequences of unpalatable prey: phytoplankton response to nutrient and predator additions. Oikos, 99, 59–68.

[ele13365-bib-0006] Bell, G. , Fugère, V. , Barrett, R. , Beisner, B. , Cristescu, M. , Fussmann, G. *et al* (2019). Trophic structure modulates community rescue following acidification. Proc. R. Soc. B, 286, 20190856. 10.1098/rspb.2019.0856 PMC6571482 31185868

[ele13365-bib-0007] de Sassi, C. & Tylianakis, J.M. (2012). Climate change disproportionately increases herbivore over plant or parasitoid biomass. PLoS ONE, 7, e40557. 22815763 10.1371/journal.pone.0040557 PMC3399892

[ele13365-bib-0008] de Boer, M.K. , Moor, H. , Matthiessen, B. , Hillebrand, H. & Eriksson, B.K. (2014). Dispersal restricts local biomass but promotes the recovery of metacommunities after temperature stress. Oikos, 123, 762–768.

[ele13365-bib-0009] Bottrell, H.H. , Duncan, A. , Gliwicz, Z.M. , Grygierek, E. , Herzig, A. , Hillbrichtilkowska, A. *et* *al* (1976). Review of some problems in zooplankton production studies. Norw. J. Zool., 24, 419–456.

[ele13365-bib-0010] Cardinale, B.J. , Srivastava, D.S. , Duffy, J.E. , Wright, J.P. , Downing, A.L. , Sankaran, M. * et* *al* (2006). Effects of biodiversity on the functioning of trophic groups and ecosystems. Nature, 443, 989–992. 17066035 10.1038/nature05202

[ele13365-bib-0011] Chapin, F.S. III , Zavaleta, E.S. , Eviner, V.T. , Naylor, R.L. , Vitousek, P.M. , Reynolds, H.L. * et* *al* (2000). Consequences of changing biodiversity. Nature, 405, 234–242. 10821284 10.1038/35012241

[ele13365-bib-0012] Chase, J.M. (2003). Community assembly: when should history matter? Oecologia, 136, 489–498. 12836009 10.1007/s00442-003-1311-7

[ele13365-bib-0013] Cohen, J.E. , Jonsson, T. & Carpenter, S.R. (2003). Ecological community description using the food web, species abundance, and body size. Proc. Natl Acad. Sci. USA, 100, 1781–1786. 12547915 10.1073/pnas.232715699 PMC149910

[ele13365-bib-0014] Culver, D.A. , Boucherle, M.M. , Bean, D.J. & Fletcher, J.W. (1985). Biomass of freshwater Crustacean zooplankton from length‐weight regressions. Can. J. Fish. Aquat. Sci., 42, 1380–1390.

[ele13365-bib-0015] De Bie, T. , De Meester, L. , Brendonck, L. , Martens, K. , Goddeeris, B. , Ercken, D. *et al* (2012). Body size and dispersal mode as key traits determining metacommunity structure of aquatic organisms. Ecol. Lett., 15, 740–747. 22583795 10.1111/j.1461-0248.2012.01794.x

[ele13365-bib-0016] Dumont, H.J. , Van de Velde, I. & Dumont, S. (1975). The dry weight estimate of biomass in a selection of Cladocera, Copepoda and Rotifera from the plankton, periphyton and benthos of continental waters. Oecologia, 19, 75–97. 28308833 10.1007/BF00377592

[ele13365-bib-0017] Eggers, S.L. , Eriksson, B.K. & Matthiessen, B. (2012). A heat wave and dispersal cause dominance shift and decrease biomass in experimental metacommunities. Oikos, 121, 721–733.

[ele13365-bib-0018] Gich, F. , Schubert, K. , Bruns, A. , Hoffelner, H. & Overmann, J. (2005). Specific detection, isolation, and characterization of selected, previously uncultured members of the freshwater bacterioplankton community. Appl. Environ. Microb., 71, 5908–5919. 10.1128/AEM.71.10.5908-5919.2005 PMC1265938 16204504

[ele13365-bib-0019] Gilman, S.E. , Urban, M.C. , Tewksbury, J. , Gilchrist, G.W. & Holt, R.D. (2010). A framework for community interactions under climate change. Trends Ecol. Evol., 25, 325–331. 20392517 10.1016/j.tree.2010.03.002

[ele13365-bib-0020] Gripenberg, S. & Roslin, T. (2007). Up or down in space? Uniting the bottom‐up versus top‐down paradigm and spatial ecology. Oikos, 116, 181–188.

[ele13365-bib-0021] Guzman, L.M. , Germain, R.M. , Forbes, C. , Straus, S. , O'Connor, M.I. , Gravel, D. * et* *al* (2019). Towards a multi‐trophic extension of metacommunity ecology. Ecol. Lett., 22, 19–33. 30370702 10.1111/ele.13162

[ele13365-bib-0022] Hahn, M.W. , Jezberova, J. , Koll, U. , Saueressig‐Beck, T. & Schmidt, J. (2016). Complete ecological isolation and cryptic diversity in *Polynucleobacter* bacteria not resolved by 16S rRNA gene sequences. ISME J., 10, 1642–1655. 26943621 10.1038/ismej.2015.237 PMC4913878

[ele13365-bib-0023] Hillebrand, H. , Durselen, C.D. , Kirschtel, D. , Pollingher, U. & Zohary, T. (1999). Biovolume calculation for pelagic and benthic microalgae. J. Phycol., 35, 403–424.

[ele13365-bib-0024] Howeth, J.G. & Leibold, M.A. (2010). Species dispersal rates alter diversity and ecosystem stability in pond metacommunities. Ecology, 91, 2727–2741. 20957966 10.1890/09-1004.1

[ele13365-bib-0025] Klug, J.L. , Fischer, J.M. , Ives, A.R. & Dennis, B. (2000). Compensatory dynamics in planktonic community responses to pH perturbations. Ecology, 81, 387–398.

[ele13365-bib-0026] Kratina, P. , Greig, H.S. , Thompson, P.L. , Carvalho‐Pereira, T.S.A. & Shurin, J.B. (2012). Warming modifies trophic cascades and eutrophication in experimental freshwater communities. Ecology, 93, 1421–1430. 22834382 10.1890/11-1595.1

[ele13365-bib-0027] Kremer, C.T. , Gillette, J.P. , Rudstam, L.G. , Brettum, P. & Ptacnik, R. (2014). A compendium of cell and natural unit biovolumes for >1200 freshwater phytoplankton species. Ecology, 95, 2984–2984.

[ele13365-bib-0028] Ledger, M.E. , Brown, L.E. , Edwards, F.K. , Milner, A.M. & Woodward, G. (2013). Drought alters the structure and functioning of complex food webs. Nat. Clim. Change, 3, 223–227.

[ele13365-bib-0029] Limberger, R. , Birtel, J. , Farias, D.D.S. & Matthews, B. (2017). Ecosystem flux and biotic modification as drivers of metaecosystem dynamics. Ecology, 98, 1082–1092. 28112404 10.1002/ecy.1742

[ele13365-bib-0030] Lindo, Z. , Whiteley, J. & Gonzalez, A. (2012). Traits explain community disassembly and trophic contraction following experimental environmental change. Glob. Change Biol., 18, 2448–2457.

[ele13365-bib-0031] Loreau, M. , Mouquet, N. & Gonzalez, A. (2003). Biodiversity as spatial insurance in heterogeneous landscapes. Proc. Natl Acad. Sci. USA, 100, 12765–12770. 14569008 10.1073/pnas.2235465100 PMC240692

[ele13365-bib-0032] Martin, T.E. & Maron, J.L. (2012). Climate impacts on bird and plant communities from altered animal‐plant interactions. Nat. Clim. Change, 2, 195–200.

[ele13365-bib-0033] McCann, K.S. , Rasmussen, J.B. & Umbanhowar, J. (2005). The dynamics of spatially coupled food webs. Ecol. Lett., 8, 513–523. 21352455 10.1111/j.1461-0248.2005.00742.x

[ele13365-bib-0034] McCauley, D.J. , Gellner, G. , Martinez, N.D. , Williams, R.J. , Sandin, S.A. , Micheli, F. * et* *al* (2018). On the prevalence and dynamics of inverted trophic pyramids and otherwise top‐heavy communities. Ecol. Lett., 21, 439–454. 29316114 10.1111/ele.12900

[ele13365-bib-0035] Myers, J.A. & Harms, K.E. (2009). Seed arrival, ecological filters, and plant species richness: a meta‐analysis. Ecol. Lett., 12, 1250–1260. 19723285 10.1111/j.1461-0248.2009.01373.x

[ele13365-bib-0036] Ockendon, N. , Baker, D.J. , Carr, J.A. , White, E.C. , Almond, R.E.A. , Amano, T. * et* *al* (2014). Mechanisms underpinning climatic impacts on natural populations: altered species interactions are more important than direct effects. Glob. Change Biol., 20, 2221–2229. 10.1111/gcb.12559 24677405

[ele13365-bib-0037] O'Connor, M.I. , Piehler, M.F. , Leech, D.M. , Anton, A. & Bruno, J.F. (2009). Warming and resource availability shift food web structure and metabolism. PLoS Biol., 7, e1000178. 19707271 10.1371/journal.pbio.1000178 PMC2723928

[ele13365-bib-0038] Petchey, O.L. , McPhearson, P.T. , Casey, T.M. & Morin, P.J. (1999). Environmental warming alters food‐web structure and ecosystem function. Nature, 402, 69–72.

[ele13365-bib-0039] Peters, R.H. & Downing, J.A. (1984). Empirical analysis of zooplankton filtering and feeding rates. Limnol. Oceanogr., 29, 763–784.

[ele13365-bib-0040] Purvis, A. , Gittleman, J.L. , Cowlishaw, G. & Mace, G.M. (2000). Predicting extinction risk in declining species. Proc. R. Soc. B, 267, 1947–1952. 10.1098/rspb.2000.1234 PMC1690772 11075706

[ele13365-bib-0041] Ramette, A. (2009). Quantitative community fingerprinting methods for estimating the abundance of operational taxonomic units in natural microbial communities. Appl. Environ. Microb., 75, 2495–2505. 10.1128/AEM.02409-08 PMC2675222 19201961

[ele13365-bib-0042] Schuldt, A. , Assmann, T. , Brezzi, M. , Buscot, F. , Eichenberg, D. , Gutknecht, J. * et* *al* (2018). Biodiversity across trophic levels drives multifunctionality in highly diverse forests. Nat. Commun., 9, 2989. 30065285 10.1038/s41467-018-05421-z PMC6068104

[ele13365-bib-0043] Shurin, J.B. (2000). Dispersal limitation, invasion resistance, and the structure of pond zooplankton communities. Ecology, 81, 3074–3086.

[ele13365-bib-0044] Soliveres, S. , van der Plas, F. , Manning, P. , Prati, D. , Gossner, M.M. , Renner, S.C. * et* *al* (2016). Biodiversity at multiple trophic levels is needed for ecosystem multifunctionality. Nature, 536, 456–459. 27533038 10.1038/nature19092

[ele13365-bib-0045] Stevens, V.M. , Whitmee, S. , Le Galliard, J.F. , Clobert, J. , Bohning‐Gaese, K. , Bonte, D. * et* *al* (2014). A comparative analysis of dispersal syndromes in terrestrial and semi‐terrestrial animals. Ecol. Lett., 17, 1039–1052. 24915998 10.1111/ele.12303

[ele13365-bib-0046] Symons, C.C. & Arnott, S.E. (2013). Regional zooplankton dispersal provides spatial insurance for ecosystem function. Glob. Change Biol., 19, 1610–1619. 10.1111/gcb.12122 23504921

[ele13365-bib-0047] Thompson, P.L. & Gonzalez, A. (2017). Dispersal governs the reorganization of ecological networks under environmental change. Nat. Ecol. Evol., 1, 0162. 10.1038/s41559-017-0162 28812626

[ele13365-bib-0048] Thompson, P.L. & Shurin, J.B. (2012). Regional zooplankton biodiversity provides limited buffering of pond ecosystems against climate change. J. Anim. Ecol., 81, 251–259. 21950456 10.1111/j.1365-2656.2011.01908.x

[ele13365-bib-0049] Tylianakis, J.M. , Didham, R.K. , Bascompte, J. & Wardle, D.A. (2008). Global change and species interactions in terrestrial ecosystems. Ecol. Lett., 11, 1351–1363. 19062363 10.1111/j.1461-0248.2008.01250.x

[ele13365-bib-0050] Ullah, H. , Nagelkerken, I. , Goldenberg, S.U. & Fordham, D.A. (2018). Climate change could drive marine food web collapse through altered trophic flows and cyanobacterial proliferation. PLoS Biol., 16, e2003446. 29315309 10.1371/journal.pbio.2003446 PMC5760012

[ele13365-bib-0051] Verreydt, D. , De Meester, L. , Decaestecker, E. , Villena, M.J. , Van der Gucht, K. , Vannormelingen, P. * et* *al* (2012). Dispersal‐mediated trophic interactions can generate apparent patterns of dispersal limitation in aquatic metacommunities. Ecol. Lett., 15, 218–226. 22221744 10.1111/j.1461-0248.2011.01728.x

[ele13365-bib-0052] Vinebrooke, R.D. , Schindler, D.W. , Findlay, D.L. , Turner, M.A. , Paterson, M. & Milis, K.H. (2003). Trophic dependence of ecosystem resistance and species compensation in experimentally acidified lake 302S (Canada). Ecosystems, 6, 101–113.

[ele13365-bib-0053] Visser, M.C. (2008). Keeping up with a warming world; assessing the rate of adaptation to climate change. Proc. R. Soc. B, 275, 649–659. 10.1098/rspb.2007.0997 PMC2409451 18211875

[ele13365-bib-0054] Voigt, W. , Perner, J. , Davis, A.J. , Eggers, T. , Schumacher, J. , Bährmann, R. * et* *al* (2003). Trophic levels are differentially sensitive to climate. Ecology, 84, 2444–2453.

[ele13365-bib-0055] Wade, R.N. , Karley, A.J. , Johnson, S.N. & Hartley, S.E. (2017). Impact of predicted precipitation scenarios on multitrophic interactions. Funct. Ecol., 31, 1647–1658.

[ele13365-bib-0056] Ward, C.L. & McCann, K.S. (2017). A mechanistic theory for aquatic food chain length. Nat. Commun., 8, 2028. 29229910 10.1038/s41467-017-02157-0 PMC5725575

[ele13365-bib-0057] Yannarell, A.C. , Kent, A.D. , Lauster, G.H. , Kratz, T.K. & Triplett, E.W. (2003). Temporal patterns in bacterial communities in three temperate lakes of different trophic status. Microb. Ecol., 46, 391–405. 12904915 10.1007/s00248-003-1008-9

[ele13365-bib-0058] Yuan, L.L. & Pollard, A.I. (2018). Changes in the relationship between zooplankton and phytoplankton biomasses across a eutrophication gradient. Limnol. Oceanogr., 63, 2493–2507. 31598005 10.1002/lno.10955 PMC6785050

