# Supplementary material for: Spatial insurance in multi‐trophic metacommunities
Source: Ecol Lett. 2019 Aug 8;22(11):1828–37. doi: 10.1111/ele.13365 (PMC6852594; doi:10.1111/ele.13365)
Supplement: Supplementary file 1 [file ELE-22-1828-s001.pdf]

Supplemental tables and figures

Table S1: Factors that influence the capacity of dispersal to facilitate species sorting and maintain diversity under environmental change. The potential for spatial insurance could vary among functional groups if they differ in these factors.

	Theoretical prediction	Reference
Availability of suitable species in the regional species pool	Colonization success of a species increases when its environmental optimum is closer to the environmental state of the patch	Thompson and Gonzalez (2017)
Dispersal rate	Colonization success increases with dispersal rate	Thompson and Gonzalez (2017)
Size of the colonizing population	Colonization success increases with the size of the colonizing population	Thompson and Gonzalez (2017)
Growth rate of the colonizing population	Rate of spread increases with population growth rate	Skellam (1951), Hastings *et al.* (2005)
Invasibility of the resident community	Negative interactions (competition, predation) with resident species can prevent successful colonization	Thompson and Gonzalez (2017)
Dependency on the presence of other species	Predators can only track environmental change if their prey is available in the novel habitat	Thompson and Gonzalez (2017)

References

Hastings, A., Cuddington, K., Davies, K.F., Dugaw, C.J., Elmendorf, S., Freestone, A. *et al.* (2005). The spatial spread of invasions: new developments in theory and evidence. *Ecol. Lett.*, 8, 91-101.

Skellam, J.G. (1951). Random dispersal in theoretical populations. *Biometrika*, 38, 196-218.

Thompson, P.L. & Gonzalez, A. (2017). Dispersal governs the reorganization of ecological networks under environmental change. *Nat. Eco. Evo.*, 1, 0162.

Table S2: Environmental conditions in the ponds and lakes used for inoculation of the mesocosms. NA: not available

	pH	Conductivity (µS cm^{-1})	O_2 (mg L^{-1})	O_2 (%)	Temperature (°C)
Pond 3 Botanical Garden	8.12	231	12.3	125	14.6
Lake Mondsee	8.15	339	11.7	107	9.5
Pond Hans-Donnenberg Park	7.50	370	7.4	73	12.8
Lake Leopoldskron	8.09	252	9.4	95	14.0
University Pond	7.52	NA	NA	NA	13.9

Table S3: Immigration ponds. At each of six immigration events we sampled water from 34-35 water bodies. Maximum distances to the mesocosm site were 10 km to the South, 22 km to the North, and 22 km to the East. The immigration pool changed slightly between immigration events when some ponds were inaccessible due to flooding or occupation by wild boar. In either mid July or early August, we measured the pH of the water bodies. NA: not available

Immigration pond	Longitude	Latitude	pH	Immigration events
Backwater Anthering 0	12°59'04"	47°52'34"	8.12	5
Backwater Anthering 1	12°59'06"	47°52'35"	7.96	6
Backwater Anthering 2	12°59'06"	47°52'32"	8.02	6
Backwater Anthering 3	12°59'17"	47°52'38"	7.77	2
Backwater Anthering 4	12°59'25"	47°52'30"	8.15	6
Backwater Anthering 5	12°59'26"	47°52'28"	7.92	6
Backwater Anthering 6	12°59'25"	47°52'39"	NA	4
Backwater Puch 1	13°05'04"	47°42'33"	7.43	6
Backwater Puch 2	13°04'53"	47°42'35"	8.00	6
Bog Botanical Garden	13°03'35"	47°47'11"	5.70	6
Bog Wasenmoos	13°15'09"	47°52'01"	4.25	6
Pond at University	13°03'32"	47°47'16"	7.60	6
Lake Eglsee, Scharfling	13°23'44"	47°47'45"	8.10	6
Lake Grabensee, West end	13°05'10"	47°58'59"	8.39	5
Lake Großegelsee	13°07'28"	47°57'43"	8.30	6
Lake Leopoldskron	13°02'11"	47°47'14"	8.77	6
Lake Lugingersee	13°02'27"	47°52'27"	8.78	6
Lake Mattsee	13°06'27"	47°58'25"	8.37	6
Lake Mitteregelsee	13°07'28"	47°57'32"	8.16	6
Lake Mondsee	13°25'15"	47°47'59"	8.20	6
Lake Obertrum	13°04'51"	47°58'07"	8.30	6
Lake Ragginger See	13°02'35"	47°52'24"	7.81	3
Lake Salzachsee 1	13°01'15"	47°49'57"	8.23	6
Lake Salzachsee 2	13°01'12"	47°50'00"	8.11	6
Lake Unteregelsee	13°07'40"	47°57'25"	7.84	5
Lake Wallersee	13°08'38"	47°54'12"	8.00	6
Pond 2, Botanical Garden	13°03'35"	47°47'13"	6.77	6
Pond 6, Botanical Garden	13°03'34"	47°47'13"	6.80	6
Pond Hans-Donnenberg Park	13°02'39"	47°47'27"	7.78	6
Pond Lichtenbuch-Rossmoos	13°28'31"	47°52'06"	6.65	6
Puddle 1 Lichtenbuch-Rossmoos	13°28'31"	47°52'06"	5.60	6
Puddle 2 Lichtenbuch-Rossmoos	13°28'38"	47°51'51"	6.80	6
River Königsseeache	13°04'31"	47°43'50"	7.84	6
River Salzach	13°04'56"	47°43'47"	8.08	6
Samer Mösl	13°04'23"	47°49'41"	5.59	6
Stream at Leopoldskron	13°02'17"	47°47'22"	7.69	6
Stream at University	13°03'40"	47°47'14"	NA	2

Table S4: Relative abundance in the immigration pool of all taxa that established in the mesocosms. Within each block and pH level, we selected the taxa that occurred in connected but not in unconnected mesocosms (i.e. the taxa that were driving the diversity response to dispersal) and calculated their total relative abundance in the immigration pool at the immigration event prior to the respective sampling date. Note that we lost the phytoplankton sample of the immigration event on day 62 and thus compared sampling day 84 with immigration event day 51. Values are averages over blocks.

	Day	pH	Total relative abundance in the dispersal pool of taxa that established locally
Phytoplankton	56	ambient	0.02
	84	ambient	0.05
	140	ambient	0.08
	56	low	0.03
	84	low	0.02
	140	low	0.17
Zooplankton	56	ambient	0.06
	84	ambient	0.03
	140	ambient	0.04
	56	low	0.07
	84	low	0.03
	140	low	0.04

Table S5: Results (i.e. P-values) of linear mixed effects models testing for effects of time, pH and dispersal (D) across sampling days 56, 84, and 140. Biomass data were ln-transformed prior to analysis, the proportion of edible phytoplankton was arcsin square root transformed. Bold font denotes P < 0.05.

	Time	pH	D	pH×Time	D×Time	pH×D	pH×D×Time
Diversity							
Zoo Diversity	< **0.001**	**0.002**	0.149	0.213	0.106	0.792	0.330
Phyto Diversity	0.193	0.054	**0.012**	0.193	0.055	**0.046**	0.092
Bac Diversity	**0.015**	**0.023**	0.231	0.130	0.244	0.467	**0.001**
Biomass and size structure							
Zoo Biomass	**0.046**	0.622	0.571	0.424	0.871	0.359	0.229
Ciliate abundance	**0.042**	0.647	**0.044**	0.472	0.054	**0.030**	0.221
Phyto biovolume	**0.034**	< **0.001**	0.610	**0.033**	0.754	0.410	0.961
Zoo:Phyto biomass	**0.016**	**0.001**	0.780	0.060	0.773	0.894	0.531
Indiv. Zoo biomass	**0.044**	0.415	0.101	**0.002**	**0.002**	0.076	**0.001**
Edible phyto	0.155	< **0.001**	0.923	0.672	0.958	0.698	0.565

D: Dispersal, Zoo: Zooplankton, Phyto: Phytoplankton, Bac: Bacteria, Edible phyto: proportion of edible phytoplankton, Indiv. Zoo biomass: mean individual zooplankton biomass

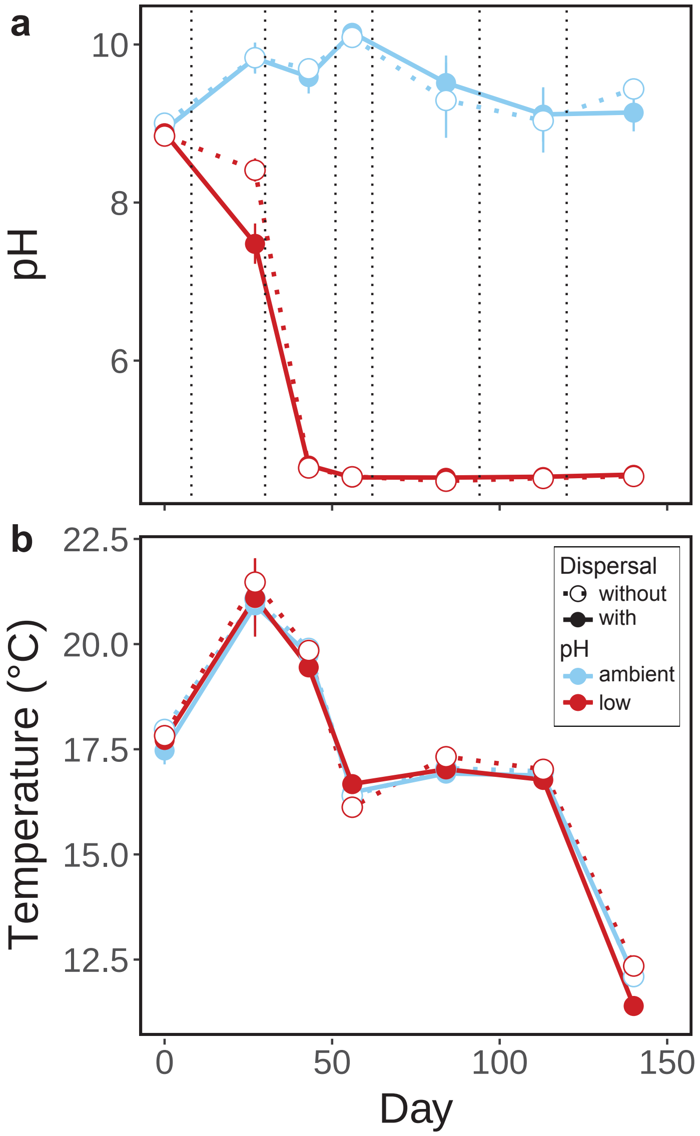

Figure S1: (a) pH and (b) water temperature in the mesocosms. Note that (a) does not show short-term flucuations in pH. In the phase of the gradual pH decline, pH increased overnight due to the buffering capacity of the water. However, once we had reached the final pH value of 4.5, we managed to keep the mesocosms at this value with only small fluctuations between pH 4.5 and 5 by regularly adding hydrochloric acid. Dotted vertical lines in (a) mark the six immigration events. Values are means ± SE, n = 4.

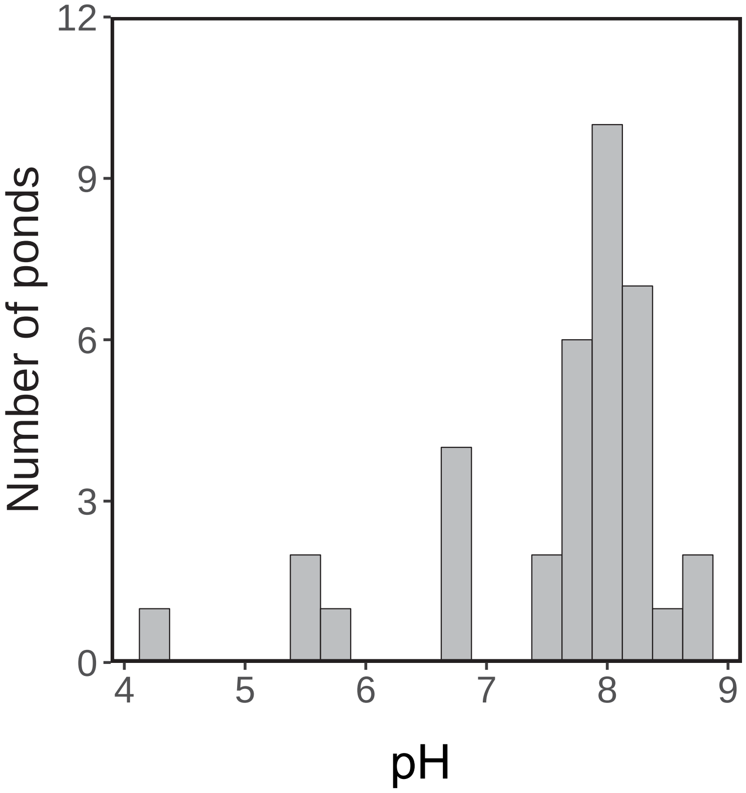

Figure S2: pH-values in the ponds and lakes used for the immigration treatment. The majority of water bodies had pH-values around 8, which is typical for the region.

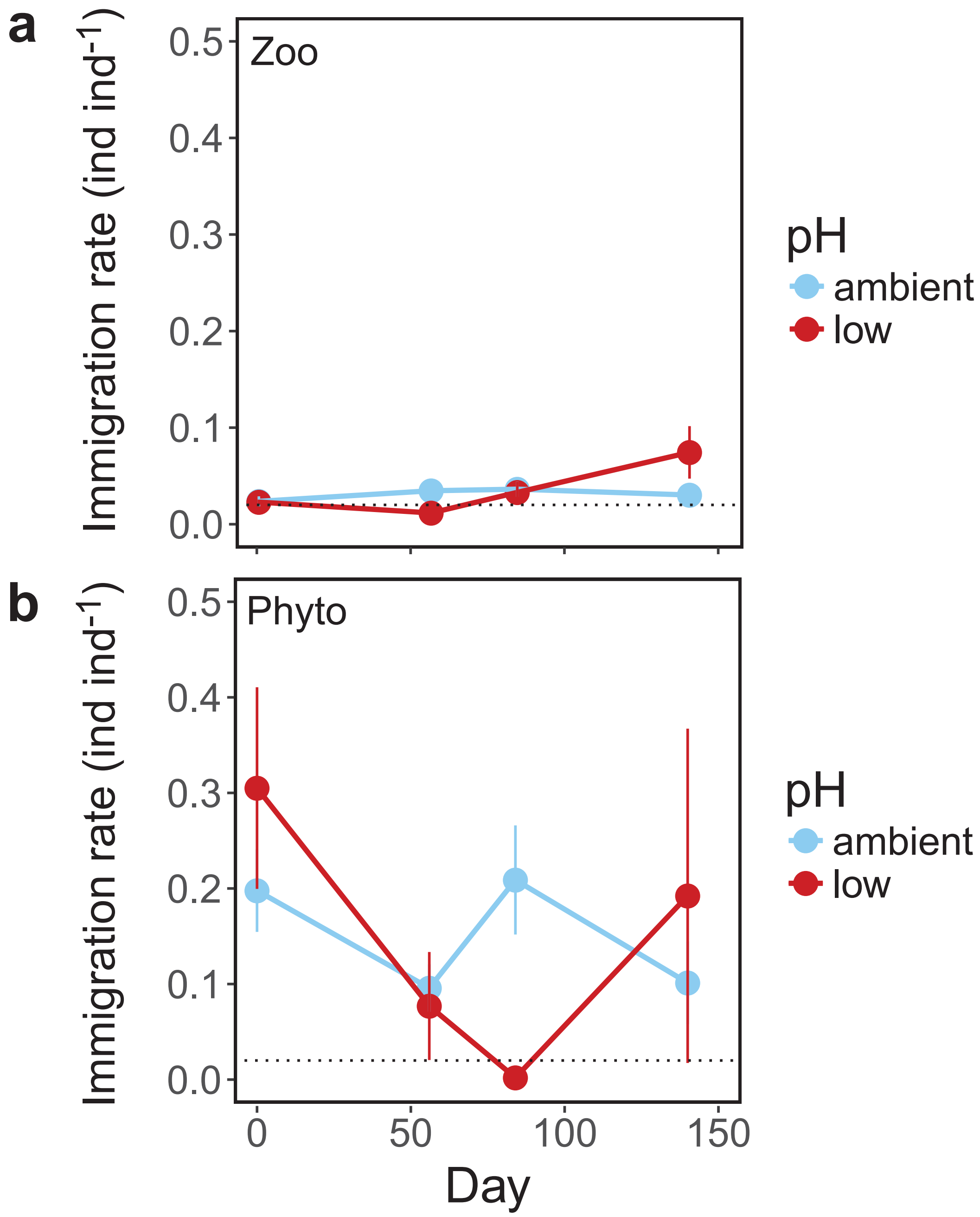

Figure S3: Immigration rate of (a) zooplankton and (b) phytoplankton. Immigration rate was calculated as the ratio of local abundance in the mesocosms to the average abundance of immigrants. The dotted line denotes the expected immigration rate of 0.02, based on 2% exchange of mesocosm water. The immigration rate did not differ between ambient and low pH on day 0 (linear mixed-effects model; zooplankton P = 0.92, phytoplankton: P = 0.23). Later in the experiment, immigration rates differed between ambient and low pH when the pH treatment affected the abundance of the resident community (marginally significant; zooplankton day 56: P = 0.018, phytoplankton day 84: P = 0.034).

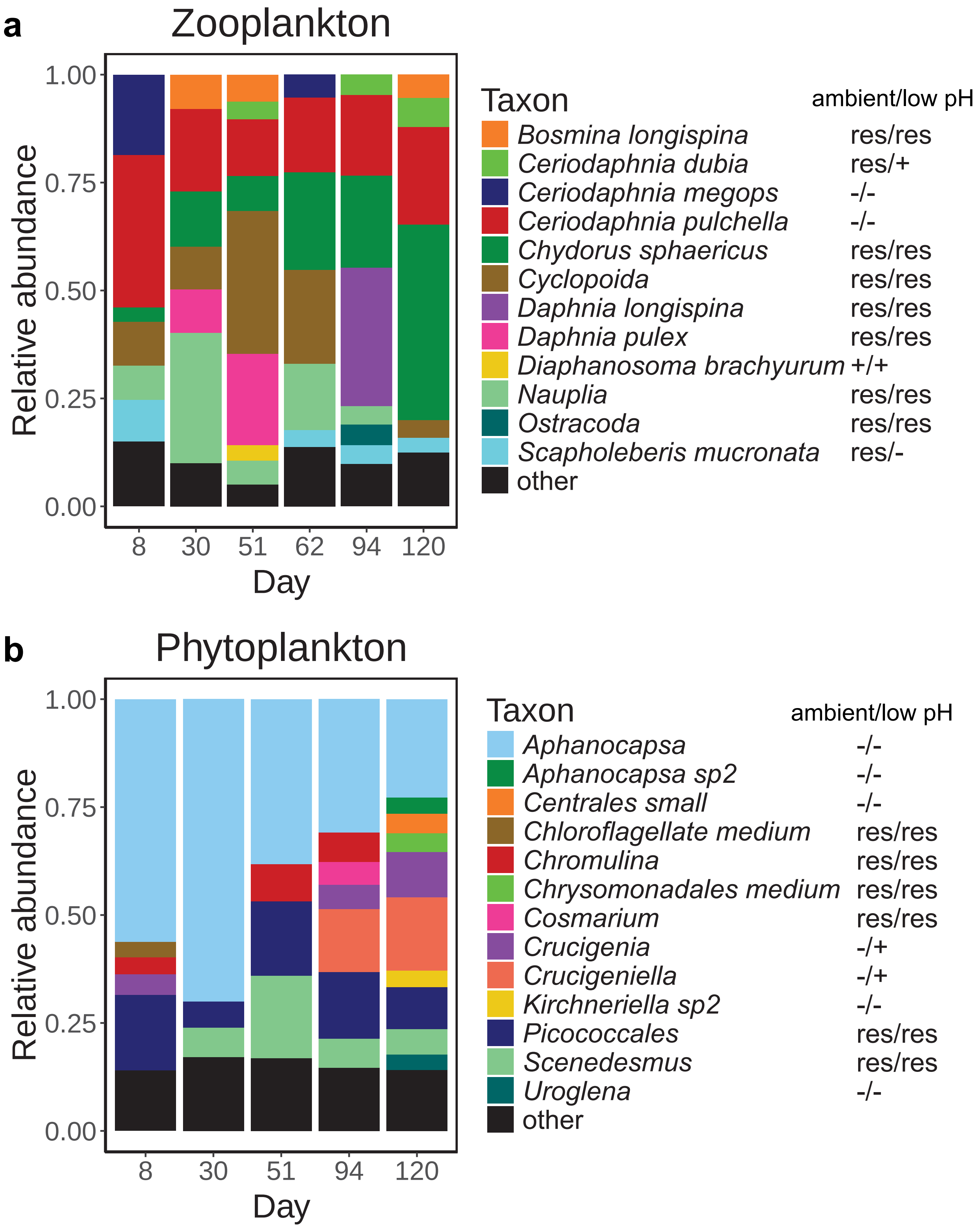

Figure S4: Composition of the dispersal pool. Relative abundance of (a) zooplankton taxa and (b) phytoplankton taxa in the dispersal pool. Note that we lost the phytoplankton sample of the immigration event on day 62. For each immigration event, all taxa with relative abundance > 0.03 are shown; all other taxa were lumped together as "other". Labels accompanying the taxa names describe whether the respective taxon was part of the resident community in unconnected mesocosms (res), established, i.e. occurred in connected but not unconnected mesocosms (+), did not establish, i.e. was never observed in any of the mesocosms (-).This information is given separately for ambient and low pH (e.g. -/+ describes a taxon that did not establish at ambient pH, but established at low pH).

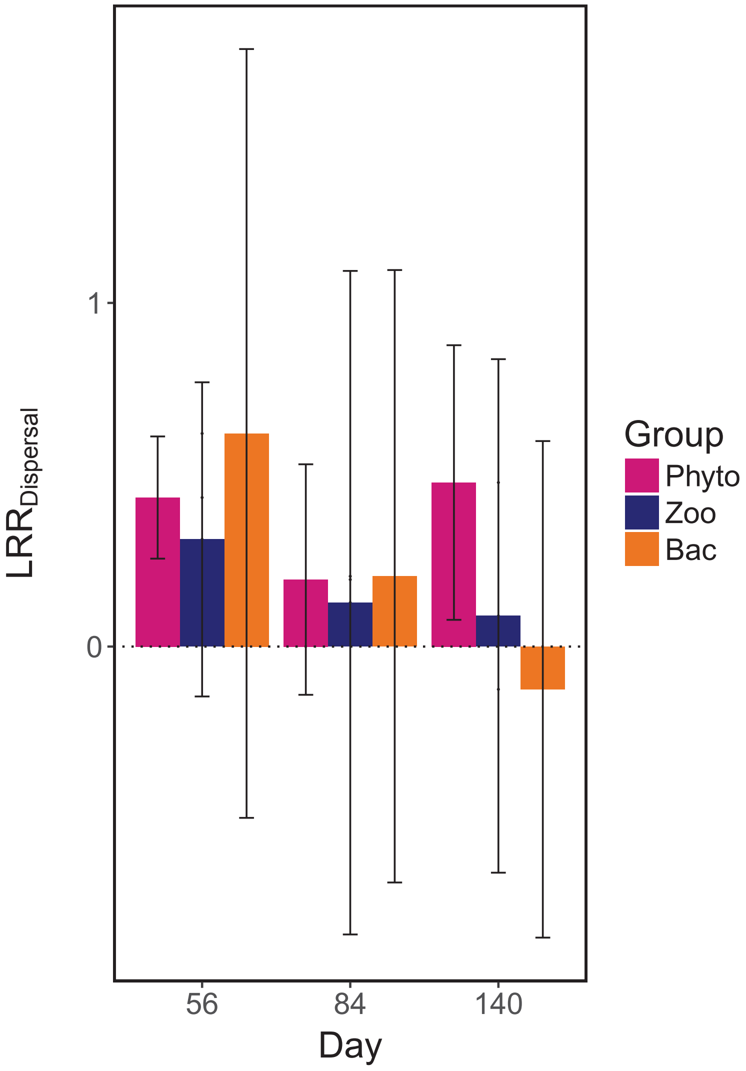

Figure S5: Effect size of dispersal on diversity at low pH. Values are log-response ratios (LRR) ± 95% confidence intervals. We calculated LRRs (i.e. ln(with dispersal/without dispersal) within each block and then averaged over the four blocks. Effect sizes are significant when the confidence interval does not include 0.

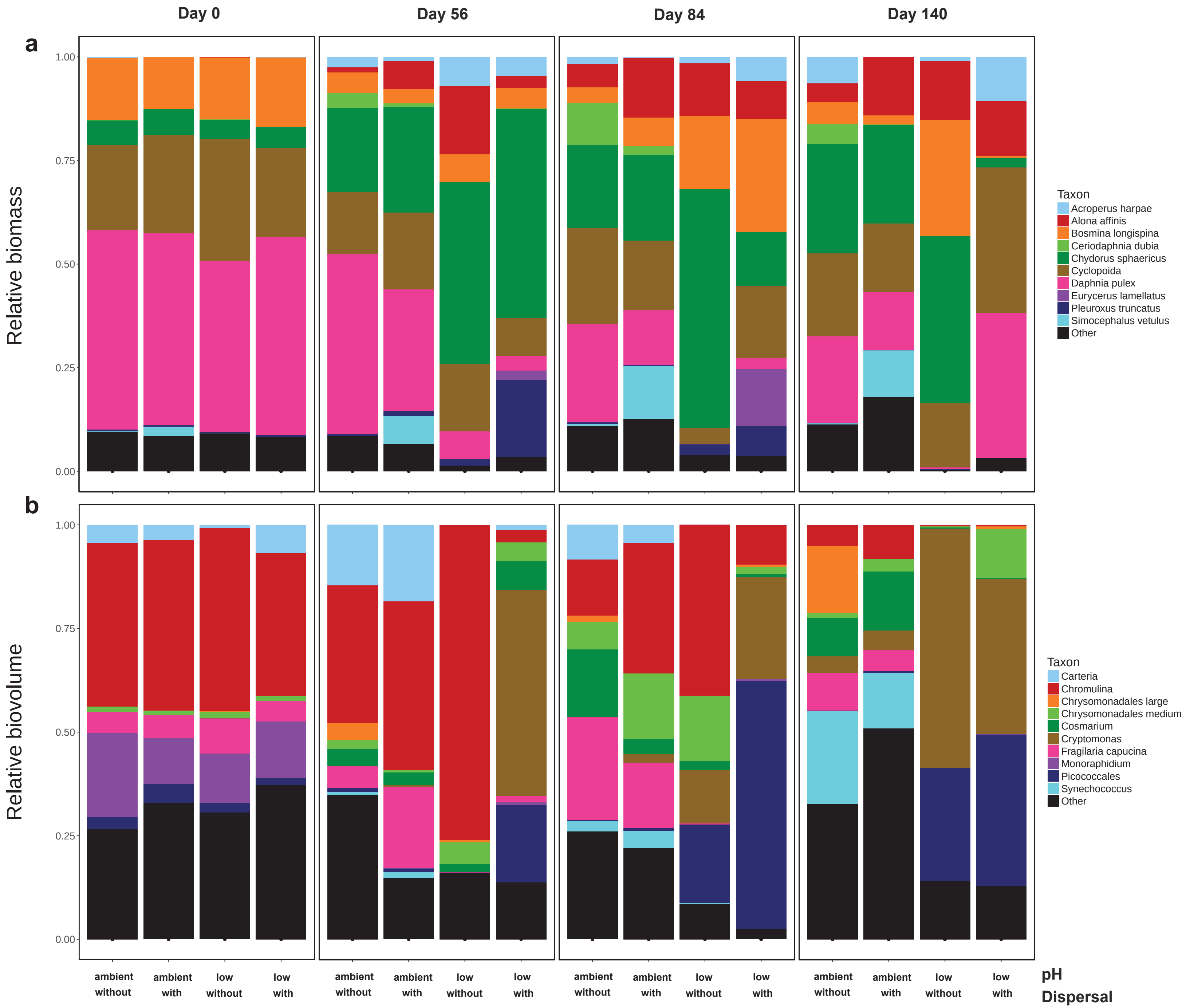

Figure S6: Treatment effects on the composition of zoo- and phytoplankton. (a) Relative biomass of macrozooplankton taxa, and (b) relative biovolume of phytoplankton taxa. Rare taxa were lumped together as "other".

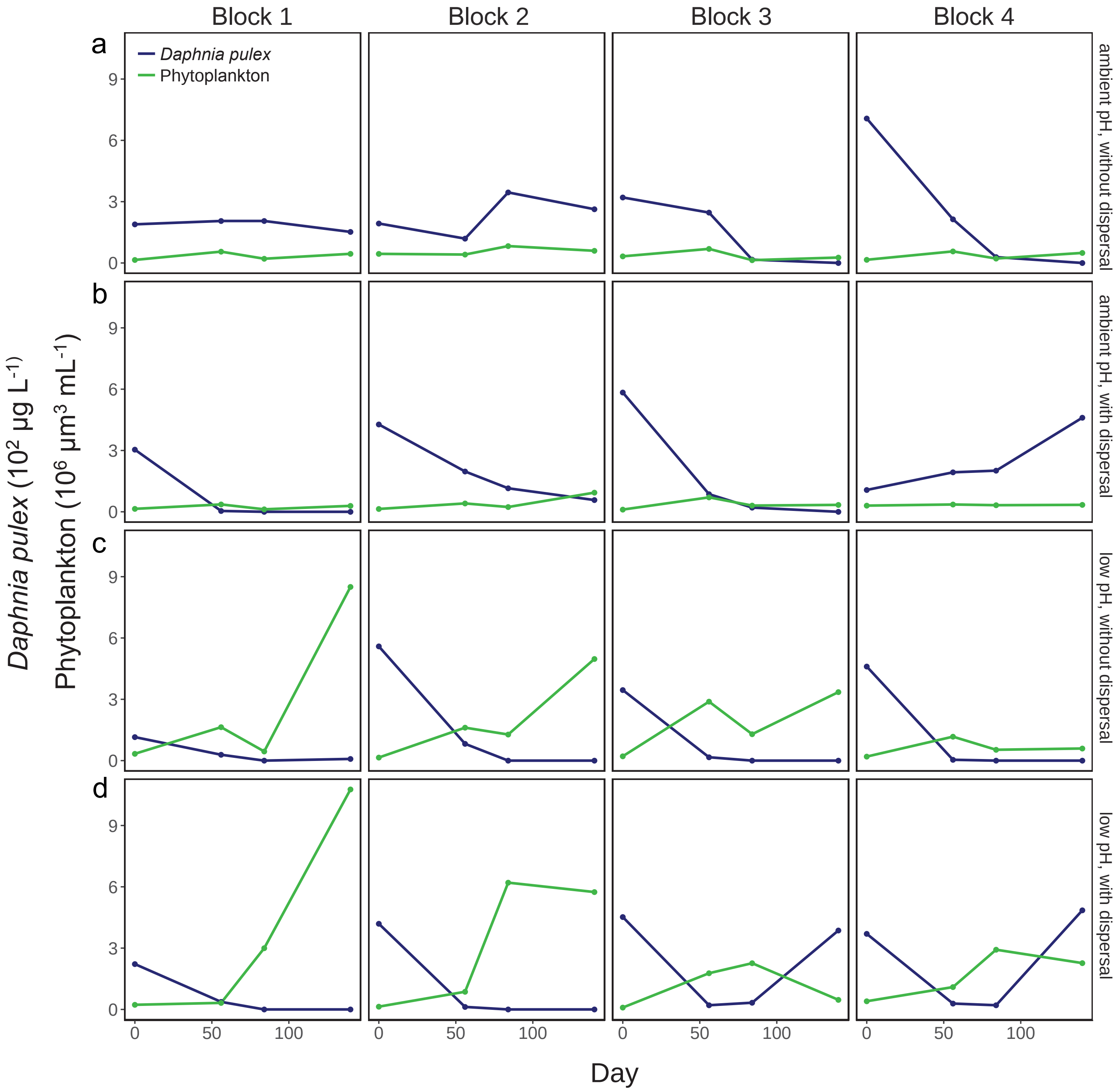

Figure S7: Biomass of *Daphnia pulex* and total phytoplankton in the 16 mesocosms. (a) ambient pH without dispersal, (b) ambient pH with dispersal, (c) low pH without dispersal, and (d) low pH with dispersal.
